# Systematic culture of central catheters and infections related to catheters in a neonatal intensive care unit: an observational study

**DOI:** 10.1038/s41598-024-59371-2

**Published:** 2024-04-15

**Authors:** Marie Mazuel, Virginie Moulier, Anne-Sophie Bourrel, Cyril Guillier, Asmaa Tazi, Pierre-Henri Jarreau, Clément Chollat

**Affiliations:** 1 Neonatal Intensive Care Unit, Assistance Publique-Hôpitaux de Paris, Robert Debré Children’s Hospital, Paris, France; 2 University Department of Psychiatry, Centre d’Excellence Thérapeutique, Institut de Psychiatrie, Centre hospitalier du Rouvray, Sotteville-lès-Rouen, France; 3 Department of Bacteriology, University Paris Cité, Assistance Publique-Hôpitaux de Paris, Cochin University Hospital, 75014 Paris, France; 4 Paediatric Intensive Care Unit, Assistance Publique-Hôpitaux de Paris, Armand Trousseau University Hospital, Sorbonne Université, Paris, France; 5 Service de Médecine et Réanimation Néonatales de Port-Royal, Hôpital Cochin, APHP centre – Université Paris Cité, Paris, France; 6 Department of Neonatal Paediatrics, APHP, Service de Néonatologie, Sorbonne Université, Hôpital Armand Trousseau, 26 Av. du Dr Arnold Netter, 75012 Paris, France; 7 Université Paris Cité, INSERM, NeuroDiderot, 75019 Paris, France; 8 Unité de Recherche Clinique, Etablissement Publique de Santé de Ville Evrard, 93332 Neuilly-sur-Marne, France

**Keywords:** Paediatric research, Bacterial infection

## Abstract

Systematic culture of the tip of central lines is performed in many neonatal intensive care units (NICUs) to guide any subsequent antibiotic therapy. The clinical relevance of this procedure is debated, given the significant bacterial contamination during its removal. We aimed to describe infections related to catheters and assess the usefulness of central catheter systematic cultures for probabilistic antibiotic therapy in cases of suspicion of catheter-related infections in a NICU. A retrospective study in a NICU included all newborn patients hospitalized with a central catheter, between January 2018, and June 2019. The main outcome measures were bacterial catheter colonization, catheter-related infection rate, and simulation-based approach to antibiotic prescription. Three hundred and seventy-five newborns, with 634 central catheters were included. There were 273 (43%) catheters that were colonized by at least one microorganism. There were 183 cases of suspected sepsis, with 31 infections definitively related to the catheter. In our simulation antibiotic prescription approach, there was no significant difference in terms of the efficacy toward the microorganism(s) involved between the probabilistic antibiotic therapies proposed by the experts and those ultimately prescribed. Performing a catheter culture only if catheter-related infection is suspected could be an alternative to routine screening.

## Introduction

Late-onset sepsis (LOS), defined as infection of a newborn occurring after the first 72 h of life, causes significant morbidity in neonatal intensive care units (NICUs)^1–5^. These infections are caused by *secondary-acquired* pathogens, whether community or nosocomial, and occur through horizontal transmission^6,7^. LOSs are distinguished based on whether they involve catheter-related infections (CRIs), which are the consequence of bacterial colonization of the prosthesis^8^. Diagnostic certainty of CRI can be complex in neonatology, especially given technical difficulties^9,10^. Although the American Society of Infectious Diseases has indicated that not performing routine culture of catheters should be the default option, unless they are removed for suspected CRI^11^, systematic culture of the central catheter tip upon its removal is performed in many NICUs to screen CRIs, either out of habit or lack of scientific evidence in neonatal care. This raises the question of the clinical relevance of systematic central catheter cultures. The main objectives of our study were to describe the rate of microbiological colonization of central catheters, the rate of LOS, and more specifically CRI, in our NICU. The secondary objective was to evaluate the effectiveness of probabilistic antibiotic therapies introduced in cases of secondary sepsis without knowledge of the result of systematic catheter cultures through a prescription simulation approach. We hypothesize that systematic culture of the tip of central catheters is of little value because it does not lead to a change in how patients are managed.

## Results

### Descriptive analysis

**Figure 1:**
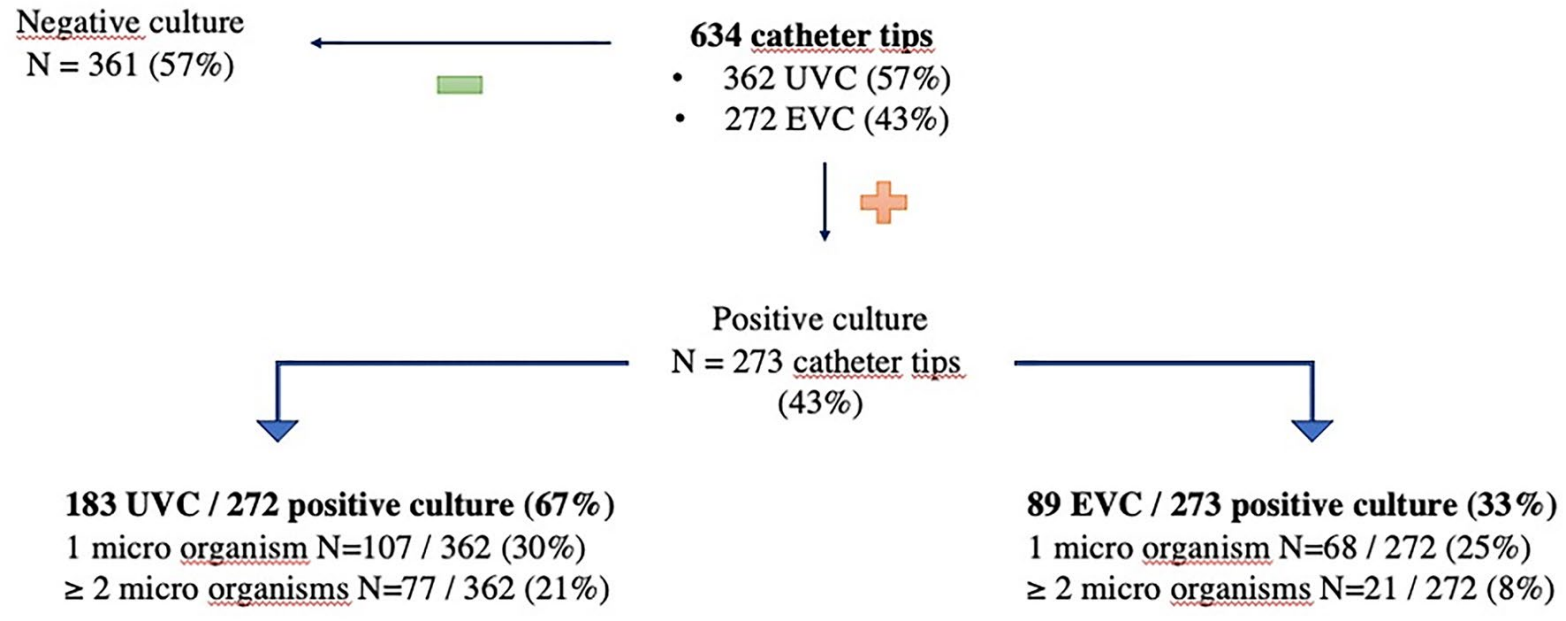
Description of catheters cultures.

A total of 375 patients were enrolled in the study. The general characteristics of the population are provided in Table 1. A total of 634 central venous catheter tips were cultured (362 umbilical venous catheter (UVC), 272 epicutaneous venous catheters (EVC); Fig. 1). The main microbial entities found on catheter cultures were coagulase-negative staphylococci (293/634, 43%, Table 2). Positive culture with two different microbial organisms recovered two coagulase-negative staphylococci in 75% of cases (67/89). A total of 183 cases of suspected sepsis were identified, of which 97 (53%) were confirmed, 61 (33.3%) were probable, and 25 (13.7%) were refuted. There were 31 CRIs and 65 probable CRIs (Table 3). Among the CRIs, UVC was involved in 52% of cases, and the pathogen recovered was mainly a coagulase-negative staphylococcus. Two CRIs (6.5%) were caused by methicillin-susceptible *Staphylococcus aureus* (MSSA), two by methicillin-resistant *S. aureus* (MRSA), three by *E. faecalis*, three by *Klebsiella pneumoniae* and three (9.7%) by *Candida* sp. In asymptomatic patients in whom catheters were systematically cultured, 97 catheter tips tested positive for coagulase-negative staphylococcus, two for MSSA, and one for *Candida*. Without treatment, the course was favorable for 88 patients, while 12 died of causes unrelated to LOS.

**Table 1:** Description of the population.

Population characteristics	Patients N = 375 n (%) Average ± SD
${\mathrm{Gestational \,age\, of\, birth}}^{{\text{a}}}$- (WG)	29.3 ± 4.6
Female	183 (48.8)
Birth weight (grams)	1304 ± 811.3
${{\text{IUGR}}}^{b}$- < 3rd percentile	54 (14.4)
Caesarean section	171 (45.6)
Late onset sepsis	183 (48.8)
Death	42 (11.2)
Causes	
Respiratory	4 (1.1)
Neurological (${{\text{HIE}}}^{{\text{c}}}$, ${{\text{IVH}}}^{{\text{d}}}$)	15 (4)
Infectious (${{\text{EOS}}}^{e}$, secondary sepsis)	19 (5.1)
Digestive	4 (1.1)
Number of catheters per patient	
1	161 (43)
2	174 (46.4)
3	29 (7.7)
4	9 (2.4)
5	2 (0.5)

^a^Gestational age, weeks of gestation.

^b^Intrauterine growth retardation.

^c^Hypoxic ischemic encephalopathy.

^d^Intraventricular hemorrhage.

^e^Early onset sepsis.

**Table 2:** Identity of the microbial entities found by catheter culture.

Microbial entities	N = 634 n (%)
Coagulase-negative* Staphylococcus*	293 (43)
*Enterococcus faecalis/faecium*	32 (5)
*Klebsiella pneumoniae*	9 (2,4)
Methicillin-susceptible* Staphylococcus aureus*	8 (1.4)
*Candida* spp*.*	6 (0.9)
*Escherichia coli*	4 (0.6)
Methicillin-resistant* Staphylococcus aureus*	3 (0.5)
*Enterobacter cloacae/aerogenes*	3 (0.5)
*Acinetobacter baumannii*	3 (0.5)
*Citrobacter freundii/koseri*	2 (3.1)
*Bacillus cereus*	1 (0.1)
*Klebsiella pneumoniae* ${ESBL}^{a}$	1 (0.1)
*Escherichia coli* ${ESBL}^{a}$	1 (0.1)
*Enterobacter cloacae/aerogenes* ${ESBL}^{a}$	1 (0.1)
*Streptococcus* sp*.*	1 (0.1)

^a^Extended-spectrum beta-lactamase.

**Table 3:** Clinical situations leading to the introduction of probabilistic antibiotic therapy.

Sepsis	Total N = 183 n (%)
${{\text{CRI}}}^{a}$	31 (16.9)
Probable ${{\text{CRI}}}^{a}$	65 (35.5)
${{\text{NEC}}}^{{\text{b}}}$	28 (15.4)
Pneumonia	31 (16.9)
Meningitis	3 (1.6)
Sepsis disproven	25 (13.7)

^a^Catheter-related infection.

^b^Necrotizing enterocolitis.

### Antibiotic prescription simulation approach

For these analyses only the first LOS was considered, which corresponds to 123 patients. *Agreement between the experts' choice of antibiotic therapy and the reference (real antibiotic therapy):*

Concordance between the probabilistic antibiotic therapy chosen by the experts blinded to the catheter culture results and the antibiotic therapy actually received was low, with a kappa coefficient of 0.347. For the coverage of the microorganisms isolated by the probabilistic antibiotic therapy chosen by the experts, compared with the coverage of the actual antibiotic therapy, Light's kappa test revealed a moderate to strong level of agreement, with a kappa coefficient of 0.601.

#### Interexpert agreement

Regarding the interexpert concordance for the choice of probabilistic antibiotic therapies blinded to the catheter culture results, there was a low level of agreement, with a kappa coefficient of 0.396. Regarding the antibiotic suitability of the microorganism that was isolated, blinded from the catheter culture, Light's Kappa test found a moderate level of agreement, with a Kappa coefficient of 0.557.

#### Coverage of the involved pathogens by the probabilistic antibiotic therapies proposed by the experts

**Figure 2:**
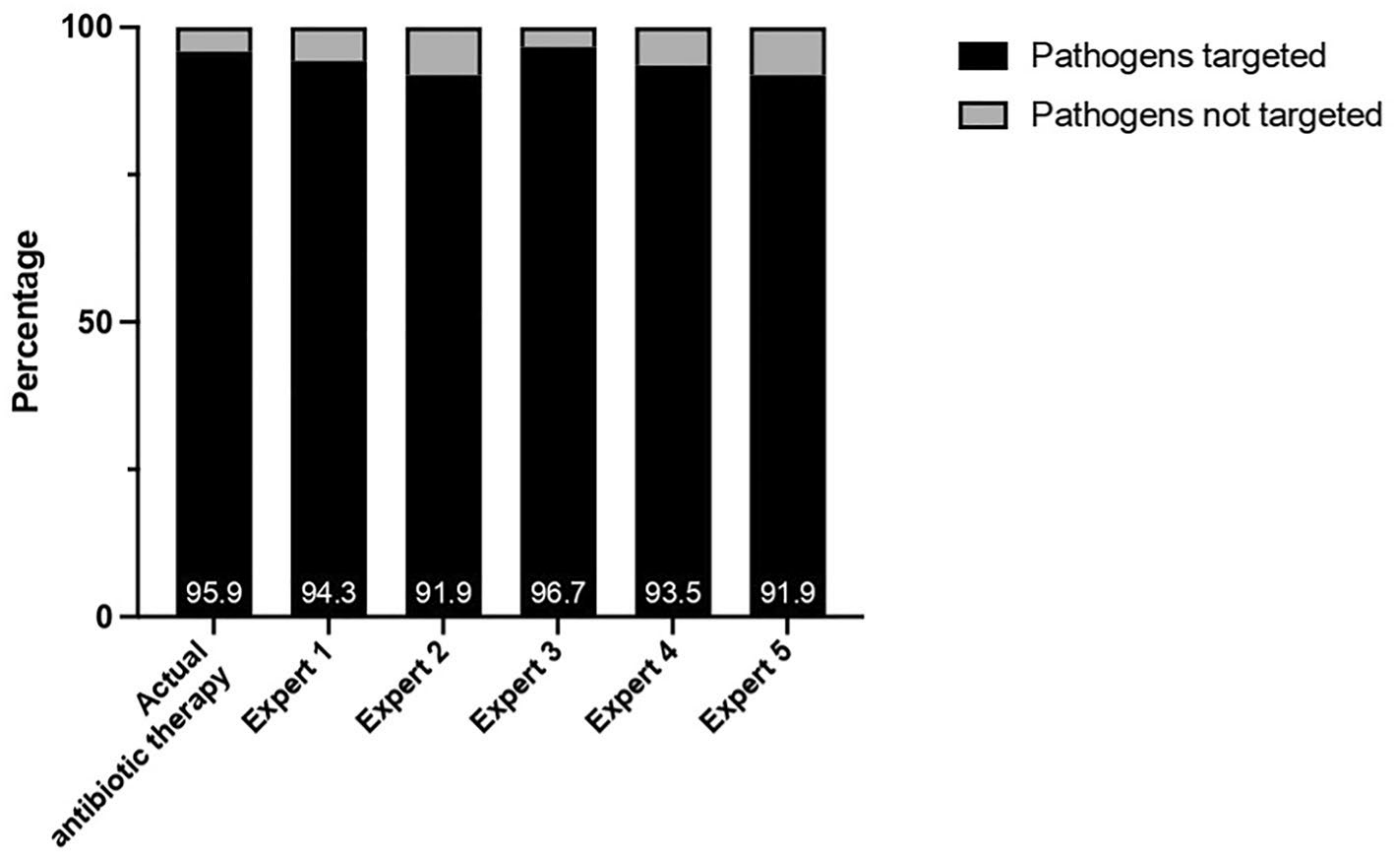
Antibiotics coverage of pathogens.

Among the 123 confirmed or probable sepsis cases, the antibiotic therapies proposed by the experts blinded to the catheter cultures were suitable in 93.7% of the situations. The ultimately prescribed probabilistic antibiotic therapies were appropriate on average for 95.9% of the sepsis cases (Fig. 2). There was no significant difference (p = 0.069) in antibiotic suitability between the antibiotic therapies prescribed by the experts and the antibiotics ultimately received.

After a case-by-case analysis, knowledge of catheter culture results knowledge of catheter culture results may have changed management in the following situations:

- Case 143: patient with noncolonized *Candida* sp*.* in birth samples, UVC positive for *Candida* sp*.* on day 5 of life, and fungemia occurred on day 13 of life, with blood cultures and a tracheal sample positive for *Candida albicans.*
- Case 166: *Enterobacter cloacae* sepsis on day 27 of life, UVC was positive for the same microorganism on day 7 of life.
- Case 288: *Candida* sp*.* colonization on day 10 of life with positive peripheral samples without positive blood culture, EVC was positive on day 7 to *Candida* sp. It should be noted that the diagnosis of sepsis with *Candida* sp*.* was not confirmed.

### Cost of routine catheter culture

Over the 18-month period, the expenses incurred by the culture of the 634 catheters amount to 25,677 euros.

## Discussion

In our NICU over 18 months, we encountered 31 CRIs, while 273/634 (43%) catheters had a positive culture. Lack of knowledge of the result of the catheter culture does not appear to modify the effectiveness of the probabilistic antibiotic cover chosen by the five experts who had been blinded to the catheter cultures.

Of the 158 confirmed or probable sepsis cases in our study, 48.1% were caused by coagulase-negative *Staphylococcus*, and for the 31 confirmed CRIs, gram-positive species were the most frequent. The distribution of pathogens found in our study was similar to that described by Stoll et al.^12^ The type of catheter involved in CRI differed from a French study: 1.3% and 14.3% CRI on UVC and EVC, respectively, versus 4.4% and 5.5%, respectively, in our study^13^. In our study, 32% of definite LOS were CRIs, whereas in the UK, the National Nosocomial Infection Surveillance Service has shown that CRIs account for 75% of the total LOS in NICUs^14^. These differences could be explained by the choice of CRI definition.

Interpreting a positive catheter culture is not straightforward. Environmental contamination of the catheter at the time of removal of the catheter cannot be excluded (coagulase-negative *Staphylococcus* and ≥ 2 microbial entities found for 43 and 32% of catheters). In addition, among asymptomatic patients who were carriers of bacterial colonization on their catheter, none had LOS. These results support therapeutic abstention in cases of colonization of the catheter by coagulase-negative staphylococci, as proposed by the study by Kitano et al.^15^.

This descriptive analysis highlights a high rate of bacterial colonization of catheters, without a direct link with the rate of LOS, including CRIs. An observational study conducted in a neonatal intensive care unit showed that a significant number of intravascular catheters were colonized with bacteria, but that only colonization of the outer surface was associated with catheter-related sepsis, representing a small proportion of colonized catheters^16^. The usefulness of systematic catheter culture was assessed by a prescription simulation approach. Interexpert concordance for the choice of probabilistic antibiotic therapy was high, as well as the effectiveness of the antibiotics on the microorganism ultimately involved.

This approach, although imperfect, allowed analysis of the choice of antibiotic therapy by fictitiously removing one of the usual key elements in decision-making in our NICU. The antibiotic suitability proposed by the experts was not significantly different from that of the antibiotic ultimately received. Indeed, the clinical and biological data available to the experts appeared to be sufficient for the choice of efficient probabilistic antibiotic therapies. Moreover, systematic culture is costly (estimated cost over the studied period amounted to EUR 25,677). If only catheters likely to be the source of LOS had been cultured (96 catheters), the cost would have been 3,888 euros. In addition, the systematic culture of catheters is a resource-intensive endeavor for bacteriology and neonatology teams.

Our study has several limitations. First, the definition of sepsis in neonatology is controversial, and many definitions are used according to the literature. We selected the definition closest to our clinical *modus operandi*^2^. The lack of consensus limits comparison of our results with other studies using a different definition. There is no consensus on the CRI definition in neonatology but the one we chose has been validated in the literature^6,17^. In fact, for a CRI to be confirmed, the pathogen must be found in both the blood and catheter culture. This definition is, consequently, dependent on the bacterial colonization of the catheter. Therefore, for this definition, it cannot be concluded that a CRI is certain when the catheter is not removed during sepsis. Moreover, our study was monocentric, reflecting our own medical practices. The retrospective collection of data based on medical records also implies a potential measurement bias. Concerning the simulation approach, the patient’s descriptive data could sometimes be insufficiently detailed, which could lead to confusing conditions for the experts.

The level of bacteriological colonization of catheter tips in the NICU is high but not necessarily associated with CRI. In the case of systematic microbiological screening of catheters at the time of removal, knowledge of the bacteriological culture does not change the suitability of antibiotics in the case of further LOS. As an alternative to routine screening, a targeted catheter culture approach could be considered in cases of removal during sepsis to support the diagnosis of CRI, which could be demonstrated in a randomized controlled trial (routine catheter culture versus targeted culture).

## Methods

### Study design

This was a retrospective, monocentric, observational study. It was approved by the Commission Nationale de l'Informatique et des Libertés, CNIL (n°1755849) and Cochin Hospital's local ethics committee. Parents were informed that the data could be used for evaluation of medical practices and that they could at any time choose to be excluded from these studies. Informed consent was obtained from all parents. All methods were carried out in accordance with relevant guidelines and French regulations.

### Setting

The tertiary maternity hospital of Port-Royal (Assistance Publique des Hôpitaux de Paris (APHP), Paris, France) handles approximately 5,500 births per year. The NICU contains 21 beds and admits approximately 500 patients each year.

### Study subjects

#### Inclusion criteria

All patients born prematurely or at term, hospitalized in the Port-Royal NICU between January 1, 2018, and June 22, 2019, and who were fitted with a central venous catheter (UVC or EVC) during their hospitalization were included in the study.

#### Data collection and definitions

Data were extracted from the hospital’s medical records. To describe our population, baseline neonatal characteristics were collected: sex, term and birth weight, and mode of delivery.

At the microbiological level, the following were collected: maternal bacterial colonization (results of vaginal samples and urinary samples if they were carried out); microbiological samples taken at birth of the patient (meconium, gastric, pharyngeal or tracheal, blood culture if it was carried out), as well as samples of systematic weekly screenings (nasal, rectal swabs). The following information about the catheters and LOS that occurred in these patients was also collected: type of catheter; date of the catheter culture; results catheter tip cultures; sepsis date; clinical signs of sepsis (hemodynamics, respiratory, neurological, digestive); biological data (leukocyte levels, platelets, CRP and PCT values); microbiological data (blood culture, lumbar puncture, pharyngeal or tracheal sampling, urine, and stool collection). For each sepsis, the probabilistic antibiotic therapy initially administered was collected.

### Catheter tip processing

Catheter tips were sent to the laboratory in sterile tubes. After the addition of 1 mL sterile water and vigorous vortexing, 100 µL were plated on Columbia agar with 5% horse blood (bioMérieux) and incubated at 37 °C in aerobic atmosphere with 5% CO2. Cultures were examined after 16 h and 44 h of incubation. Bacterial and yeast colonies were identified using MALDI-TOF mass spectrometry (BRUKER DALTONICS). Catheter colonization was defined as a culture positive with ≥ 103 colony forming units; otherwise, catheter tips were considered contaminated.

### Definition of sepsis

Sepsis was classified into "confirmed" sepsis (evidence level number 1), "probable" (evidence level number 2 or 3), or "disproved" (no validated level of evidence) according to the definition of the GAIA (*Global Alignment of Immunisation Safety Assessment in Pregnancy*): *Neonatal Infections Working Group* (Data Supplements 1)^2^.

### Definition of CRI

Sepsis (as defined above) was considered catheter-related when the tip of the catheter was tested positive for a pathogen identical to that found in a blood culture collected within 72 h before or after catheter removal, in the presence of no other clinical starting point. Sepsis was considered suspicious of being related to the catheter (probable CRI) if no other starting point was identified and sepsis occurred within 72 h before or after catheter removal^10,15^.

### Antibiotics prescription simulation approach

To analyze the clinical relevance of systematic culture of central catheters, three neonatologists and two bacteriologists were asked to propose a probabilistic antibiotic therapy for each first LOS, CRI or not, blinded to the result of the culture of the removed catheter(s) before antibiotic therapy. The experts chose from a predefined list of antibiotic combinations (Data Supplement 2). Of note, in this unit, if the patient had a central or peripheral line, antibiotic treatment included vancomycin and gentamicin. If NEC was suspected, cefotaxime and metronidazole were added. Other antibiotic approach was possible depending on patient colonization or clinical symptoms. We have added this precision in the methods section. The experts had access to the following information:

- Term and birth weight;
- Maternal and patient colonization;
- Date of occurrence of secondary sepsis;
- Clinical manifestations (hemodynamic, respiratory, neurological, and digestive);
- Biological data (leukocyte levels, CRP values);
- Presence or absence of a catheter at the time of sepsis;
- History of sepsis and the causative pathogen involved.

The antibiotic therapies proposed by the experts were compared with the antibiotic therapies ultimately received by the patients. In a second step, the effectiveness of the probabilistic antibiotic therapies proposed by the experts was compared to that of the therapies ultimately prescribed. For these analyses, when the same patient had two sepsis, we chose to take into account only the first sepsis. Indeed, we considered that the two sepsis of the same patient were nonindependent variables.

### Cost of routine catheter culture

We sought to evaluate the cost of routine catheter tip culture. The unit cost of catheter culture is 40.5 euros.

### Statistical analysis

For the descriptive analysis, the continuously collected variables are expressed as averages with their standard deviations, and the categorical variables are expressed as numbers and percentages. To assess the interobserver consistency, Light's Kappa test was used. This test consisted of performing a Cohen’s Kappa test for each possible pair of observers (in this case 10 pairs) and then averaging the Kappa tests obtained. Regarding the two categorical variables (choice of treatment and coverage), Light's kappa was performed to assess the interobserver agreement, between the five clinicians and between each clinician and the reference method. Light's kappa equaled the average of all possible combinations of bivariate kappa values between raters. Cochrane’s Q test was used to test for differences in coverage frequencies between the antibiotic therapies prescribed by the experts and the antibiotics ultimately received. The analyses were conducted using SPSS, version 28 (IBM, Armonk, NY, www.ibm.com/support/pages/downloading-ibm-spss-statistics-28).

### Ethics approval and consent to participate

This was a retrospective, monocentric, observational study. It was approved by the Commission Nationale de l'Informatique et des Libertés, CNIL (n°1755849) and Cochin Hospital’s local ethics committee. Parents were informed that the data could be used for evaluation of medical practices and that they could at any time choose to be excluded from these studies. Informed consent was obtained from all parents. All methods were carried out in accordance with relevant guidelines and French regulations.

### Supplementary Information

Supplementary Information.

## Data Availability

The datasets used and analysed during the current study are available from the corresponding author on reasonable request.

## Abbreviations

CRI: Catheter-related infection
EVC: Epicutaneous venous catheter
LOS: Late-onset sepsis
MRSA: Methicillin-resistant *Staphylococcus aureus*
MSSA: Methicillin-susceptible *Staphylococcus aureus*
NICU: Neonatal intensive care unit
UVC: Umbilical venous catheter
