# Supplementary material for: Systematic culture of central catheters and infections related to catheters in a neonatal intensive care unit: an observational study
Source: Sci Rep. 2024 Apr 15;14:8647. doi: 10.1038/s41598-024-59371-2 (PMC11018835; doi:10.1038/s41598-024-59371-2)
Supplement: Supplementary file 1 — Supplementary Information. [file 41598_2024_59371_MOESM1_ESM.pdf]

SUPPLEMENTARY DATA

Data supplement 1: The three levels of diagnostic evidence of the GAIA group.

Level 1

- Organism recognized as pathogen* and isolated by a validated method in a normally sterile fluid**
- If an organism recognized as non-pathogenic is isolated from a blood culture, Level 1 blood cultures taken from two different sites or at two different times, AND 1 criterion of Level 2

Level 2

- Criteria that do not confirm Level 1

  AND

- Three criteria among:
  - HR > 180 or < 100/min
  - T°>37.5°C or < 35.5°C
  - Apnea***/ increase in oxygen requirements or of the ventilatory parameters
  - Hypotonia/ Irritability
  - Abdominal distension
  - Cuteneous palor/ Hypotension
  - Leukocytes < 4000 or > 20 000 /mm^3
  - Platelets < 100 000/mm^3
  - Metabolic acidosis BE < - 10 mmol/L
  - CRP or PCT above local norms

Level 3

- Criteria that do not confirm Levels 1 and 2

  AND

- Two criteria among:
  - T°>37.5°C or < 35.5°C
  - Convulsions
  - Tachypnea, cyanosis, signs of shortness of breath
  - Altered tonicity
  - Difficulty feeding

** : confers next Table*
***: blood, urine collected by sampling, pleural fluid, synovial fluid, ascites, broncho alveolar lavage, bone biopsy*
****: apnea> 20 seconds*

List of agents recognized as pathogenic and non-pathogenic.

**Agents recognized as pathogens**	**Non-pathogenic agents that can cause opportunistic infections in the neonatal period**
**BACTERIA**	
Acinetobacter sp.	*Bacillus sp. (other than cereus)*
Bacillus cereus	*Bacteroides sp.*
Bordetella sp.	*Coagulase negative staphylococci*
Citrobacter sp.	*Corynebacterium sp.*
Clostridium sp.	*Diphtheroids*
Enterococcus sp.	*Micrococcus sp.*
Enterobacter sp.	*Propionibacterium sp.*
Escherichia coli	*Peptococcus sp.*
Haemophilus sp.	*Peptostreptococcus sp.*
Klebsiella sp.	*Streptococcus sp.*
Lactobacillus sp.	
Listeria monocytogenes	
Mycobacterium sp.	
Morganella organii	
Neisseria sp.	
Nocardia sp.	
Pantoea sp.	
Proteus sp.	
Providencia sp.	
Pseudomonas sp.	
Salmonella sp.	
Shigella sp.	
**FUNGI**	
Aspergillus sp.	
Candida sp.	
Cryptococcus sp.	

Data supplement 2: Antibiotic therapies provided by the experts to respond to clinical vignettes.

0 = Vancomycin Cefotaxime Gentamicin
1 = Vancomycin Gentamicin
2 = Meropenem Gentamicin
3 = Meropenem Vancomycin Gentamicin
4 = Vancomycin Cefotaxime Gentamicin Metronidazole
5 = Meropenem Vancomycin Gentamicin Metronidazole
6 = Vancoycine
7 = Cefotaxime Gentamicin
8 = Flagyl
9 = Cefotaxime Metronidazole
10 = Amoxicillin Gentamicin
11 = Vancomycin Gentamicin Ciprofloxacin
12 = Cefotaxime Gentamicin Metronidazole
13 = Micafungin/Triflucan
14 = Vancomycin Meropenem Amiklin
15 = Amoxicillin Vancomycin Gentamicin
16 = Fosfomycin Meropenem Vancomycin Amiklin
17 = Ceftazidime Amiklin
18 = Clamoxyl Cefotaxime Gentamicin
19 = Ceftazidime Gentamicin
20 = Vancomycin Rifampicin Gentamicin
21 = Vancomycin Cefotaxime Gentamicin Micafungin/Fluconazole
22 = Vancomycin Cefotaxime Gentamicin Metronidazole Micafungin/Fluconazole
23 = Vancomycin Micafungin/Fluconazole
24 = Meropenem Vancomycin Gentamicin Micafungin/Fluconazole
